# IBDkb: an AI-enhanced integrative knowledge base for inflammatory bowel disease research and drug discovery

**DOI:** 10.1093/database/baag038

**Published:** 2026-07-10

**Authors:** Liwen Tao, Shiqing Shi, Ruixin Zhu, Zhuo Liu, Bin Yang, Lei Liu, Wanning Chen, Qian Long, Na Jiao, Guoqing Zhang, Pingping Xu, Dingfeng Wu

**Affiliations:** Shanghai Key Laboratory of Maternal Fetal Medicine, Shanghai Institute of Maternal-Fetal Medicine and Gynecologic Oncology, Clinical and Translation Research Center, Shanghai First Maternity and Infant Hospital, School of Life Sciences and Technology, Tongji University, 1239 Siping Road, Shanghai 200092, P. R. China; Shanghai Key Laboratory of Maternal Fetal Medicine, Shanghai Institute of Maternal-Fetal Medicine and Gynecologic Oncology, Clinical and Translation Research Center, Shanghai First Maternity and Infant Hospital, School of Life Sciences and Technology, Tongji University, 1239 Siping Road, Shanghai 200092, P. R. China; Shanghai Key Laboratory of Maternal Fetal Medicine, Shanghai Institute of Maternal-Fetal Medicine and Gynecologic Oncology, Clinical and Translation Research Center, Shanghai First Maternity and Infant Hospital, School of Life Sciences and Technology, Tongji University, 1239 Siping Road, Shanghai 200092, P. R. China; State Key Laboratory of Genetic and Development of Complex Phenotypes, Fudan Microbiome Center, School of Life Sciences, Fudan University, 220 Handan Road, Shanghai 200438, P. R. China; Shanghai Southgene Technology Co., Ltd, 351 Guo Shoujing Road, Shanghai 201203, China; Shanghai Key Laboratory of Maternal Fetal Medicine, Shanghai Institute of Maternal-Fetal Medicine and Gynecologic Oncology, Clinical and Translation Research Center, Shanghai First Maternity and Infant Hospital, School of Life Sciences and Technology, Tongji University, 1239 Siping Road, Shanghai 200092, P. R. China; Shanghai Key Laboratory of Maternal Fetal Medicine, Shanghai Institute of Maternal-Fetal Medicine and Gynecologic Oncology, Clinical and Translation Research Center, Shanghai First Maternity and Infant Hospital, School of Life Sciences and Technology, Tongji University, 1239 Siping Road, Shanghai 200092, P. R. China; Shanghai Key Laboratory of Maternal Fetal Medicine, Shanghai Institute of Maternal-Fetal Medicine and Gynecologic Oncology, Clinical and Translation Research Center, Shanghai First Maternity and Infant Hospital, School of Life Sciences and Technology, Tongji University, 1239 Siping Road, Shanghai 200092, P. R. China; State Key Laboratory of Genetic and Development of Complex Phenotypes, Fudan Microbiome Center, School of Life Sciences, Fudan University, 220 Handan Road, Shanghai 200438, P. R. China; National Genomics Data Center& Bio-Med Big Data Center, Chinese Academy of Sciences Key Laboratory of Computational Biology, Shanghai Institute of Nutrition and Health, Chinese Academy of Sciences, 500 Caobao Road, Shanghai 200031, P. R. China; Department of Colorectal Surgery, Zhongshan Hospital, Fudan University, 220 Handan Road, Shanghai 200032, P. R. China; Shanghai Key Laboratory of Maternal Fetal Medicine, Shanghai Institute of Maternal-Fetal Medicine and Gynecologic Oncology, Clinical and Translation Research Center, Shanghai First Maternity and Infant Hospital, School of Life Sciences and Technology, Tongji University, 1239 Siping Road, Shanghai 200092, P. R. China

## Abstract

IBDkb (Inflammatory Bowel Disease Knowledge Base; https://www.biosino.org/ibdkb) is a freely accessible, integrated web-based platform that systematically curates and harmonizes multi-source data related to inflammatory bowel disease (IBD). To address the fragmentation and therapeutic gaps in existing specialized resources, IBDkb establishes a unified framework featuring advanced full-text search, interactive visualizations, cross-module knowledge graphs, and AI-powered utilities for real-time literature retrieval, trend analysis, text/PDF interpretation, and domain-specific conversational assistance. The platform currently integrates 98 453 research articles, 3390 clinical trials, 200 investigational drugs, 200 606 bioactive compounds, 103 therapeutic targets, 77 experimental models, 12 pathogenesis summaries, and 15 treatment strategies. These integrated tools facilitate efficient exploration of complex associations among drugs, targets, trials, and mechanisms, thereby accelerating hypothesis generation and translational research in IBD. The platform is openly available without registration and supports data downloads. A case study on structure-aware drug comparison further demonstrates its utility in facilitating cross-disease drug repositioning hypotheses.

## Introduction

Inflammatory bowel disease (IBD) is a chronic and complex inflammatory disorder f the gastrointestinal tract, comprising two main conditions: Crohn’s disease (CD) and ulcerative colitis (UC) [1]. It is characterized by a lifelong, relapsing-remitting course with symptoms such as diarrhea and oabdominal pain [2, 3], and is associated with an increased risk of colorectal cancer [4]. The necessity for long-term treatment and ongoing clinical monitoring substantially impairs patients’ quality of life. Owing to its increasing incidence and prevalence worldwide, IBD now affects millions and represents a growing global public health challenge with substantial clinical and socioeconomic burdens [5, 6].

IBD arises from a multifactorial etiology involving intricate interactions among genetic susceptibility, environmental exposures, immune dysregulation, and alterations in the gut microbiota [7]. Traditional management has relied on broad anti-inflammatory and immunosuppressive therapies, including aminosalicylates, corticosteroids, occasionally thiopurines, and antibiotics for mild to moderate IBD. For moderate to severe IBD, the introduction of biologic agents such as anti-tumour necrosis factor (TNF) monoclonal antibodies has revolutionized IBD treatment [1]. However, treatment efficacy remains limited; for instance, only ~30%–50% of patients achieve clinical and mucosal remission with anti-TNF therapy. Consequently, emerging therapeutic strategies are being explored to more precisely target IBD pathogenesis. These include novel biologics (e.g. anti-IL-12/23, anti-α4β7/αEβ7 agents), small-molecule inhibitors such as Janus kinase (JAK) inhibitors, as well as cell- and microbiome-based therapies [8]. Collectively, these advances underscore the shift toward precision medicine in IBD.

Concurrently, advances in high-throughput technologies and clinical informatics have generated increasingly complex and multi-dimensional datasets in IBD research, evolving from single-omics profiles to integrative multi-omics frameworks. Study designs have also expanded from single-centre cohorts to multi-centre cohorts and specialized populations [9–11]. While these advances have greatly expanded the scope of IBD research, they have also resulted in substantial data heterogeneity and fragmentation across studies and data types. To address these challenges, several specialized databases have been developed, such as the IBD Exomes Browser for genetic variant exploration [12], the Inflammatory Bowel Disease Multi’omics Database (IBDMDB) [13], the Inflammatory Bowel Diseases Integrated Resources Portal (IBDIRP) [14], both of which focus on multi-omics data, the Ulcerative Colitis Database (UCDB) [15], the IBD database (IBDDB) [16], and the IBDB [17], which curate IBD-associated genes, as well as transcriptome-centred resources such as IBDTransDB [18] and scIBD for single-cell meta-analyses [19].

Despite their individual utility, these resources differ markedly in scope, update frequency, data granularity, and analytical functionality. Crucially, most are primarily descriptive and omics-centric, with limited support for integrating therapeutic knowledge, such as systematic curation of IBD-related drugs, treatment regimens, clinical trial evidence, experimental models, and drug-target relationships. Standardized integration and cross-linking among genomic findings, clinical trials, therapeutic strategies, and drug development-relevant resources remain largely lacking.

This fragmentation hinders data interoperability, sharing and downstream reuse, particularly in the context of IBD drug discovery, mechanism-driven therapeutic development, and AI-assisted drug design [20]. Therefore, there is an urgent need for an integrated, multi-level knowledge base that bridges molecular mechanisms, pharmacological interventions, clinical evidence, and experimental models to directly support IBD therapeutic research and drug development pipelines.

To bridge these gaps, we developed IBDkb, a comprehensive web-based knowledge base that integrates multi-source IBD-related data with manual curation, advanced visualization, and AI-enhanced tools to support translational research and drug development. To clarify the specific functionalities enabled by IBDkb relative to existing IBD-related resources, we summarized a direct feature-by-feature comparison of representative databases in Table S1.

## Materials and methods

### Dataset collection and curation

**Figure 1:**
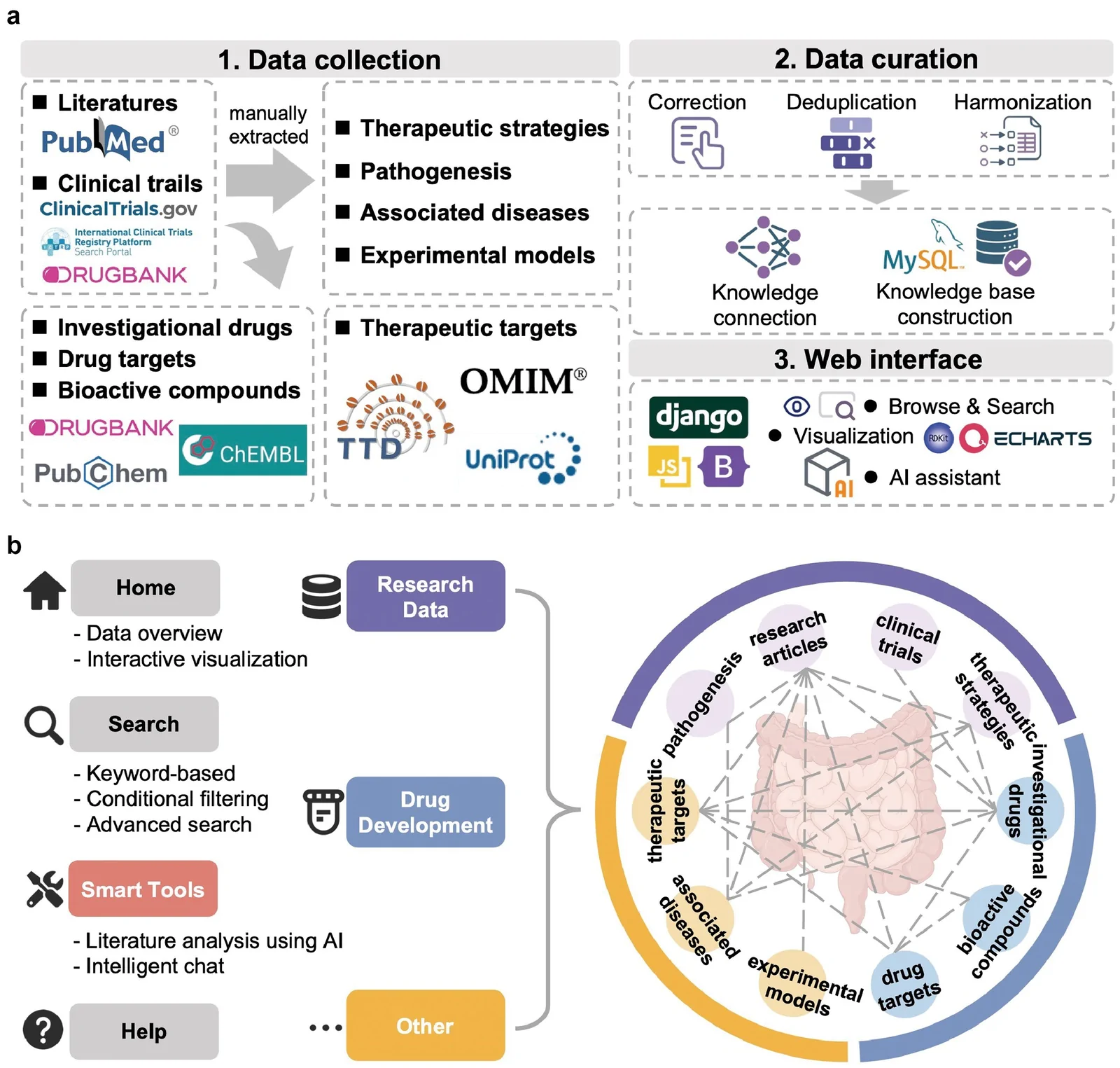
The framework and available function modules of IBDkb. (a) Schematic workflow illustrating the construction of the IBDkb knowledge base. (b) System architecture and core functionalities of the IBDkb platform.

IBDkb integrates data from diverse public sources, including research articles, clinical trials, drug information, therapeutic targets, bioactive compounds, and experimental models (Fig. 1a). As of 10 January 2026, IBDkb comprises 98 453 annotated research articles, 3390 clinical trials, 200 investigational drugs, 1404 drug targets, 200 606 bioactive compounds, 77 experimental models, 103 IBD-specific therapeutic targets, 12 pathogenesis records, and 15 therapeutic strategy records. Literature records were retrieved from PubMed using keyword-based searches and automated queries via the Python Entrez toolkit. The initial literature collection focused on articles published within the past two decades, using core terms, including ‘inflammatory bowel disease’, ‘Crohn’s disease’, and ‘ulcerative colitis’, as well as their relevant synonyms and MeSH terms. This scope was used for the initial database construction, whereas newly published articles will be continuously incorporated during quarterly updates to maintain the currency of the literature module. For each publication, the title, abstract, PubMed ID (PMID), journal name, and publication date were extracted. Clinical trial information was collected from internationally recognized registries, including ClinicalTrials.gov (https://clinicaltrials.gov), the World Health Organization’s International Clinical Trials Registry Platform (https://trialsearch.who.int), and DrugBank [21], covering essential details such as study design, recruitment status, enrollment criteria, and trial phase. From the collected research articles, IBD-related therapeutic strategies, pathogenesis, and associated diseases were manually extracted. Investigational drugs were compiled from published studies, registered clinical trials, and the DrugBank database. Drug-target relationships were derived from DrugBank [21] and PubChem [22], and bioactive compounds were retrieved from ChEMBL [23]. Data on therapeutic targets were gathered from the Online Mendelian Inheritance in Man database [24], the Therapeutic Target Database [25], and UniProt [26]. In this context, ‘drug targets’ refer to the pharmacological targets or downstream molecular targets associated with the collected drugs, regardless of whether these targets are specifically implicated in IBD. In contrast, ‘therapeutic targets’ refer to disease-relevant targets with reported associations with IBD pathogenesis or therapeutic intervention. Information on experimental models, pathogenesis, and therapeutic strategies was manually extracted and annotated from key publications. For manually curated modules, entries were standardized using predefined fields, harmonized terminology, consistent disease and intervention naming, and traceable literature evidence to ensure consistency across records.

To ensure data quality and consistency, we implemented a multi-step curation pipeline, including manual correction, deduplication, and format harmonization. Duplicate entries were removed using custom Python scripts based on unique identifiers and title/name matching. Ambiguous records were reviewed by domain experts. Data from heterogeneous sources were standardized into a unified structure. To ensure data currency and long-term sustainability, IBDkb is scheduled to be updated quarterly.

### Platform development and implementation

The web interface was developed using the Django framework (v5.1.2) with Python (v3.11.13), enabling a clear separation of system modules for easier feature expansion and maintenance. Data are managed using a MySQL database (v9.5.0) to support efficient storage and retrieval. The front-end uses standard Hypertext Markup Language for content rendering, complemented by Bootstrap for responsive styling, and JavaScript for interactivity. Client-server communication is handled through asynchronous AJAX calls to the back-end API. We applied RDKit (v2023.09.1) to support chemical structure visualization for drugs and compounds. For data analytics and interactive charts, the platform incorporates ECharts (v5.4.3).

The IBDkb platform is enhanced with artificial intelligence (AI) capabilities. Specifically, ERNIE Bot (ernie-3.5–8K; Baidu, Inc), a large language model, was selected as the AI engine for its strong performance in Chinese and English biomedical text comprehension and its accessible API. It powers intelligent literature analysis and conversational interaction. In addition, we developed a global IBD AI assistant based on a retrieval-augmented generation (RAG) framework, which retrieves evidence from the IBDkb knowledge corpus to provide grounded, context-aware responses. The platform is hosted on stable national servers with regular backups to ensure long-term availability. The platform further enables downloading of complete data resources, customized filtered results, and processed AI-ready datasets. AI-ready datasets are defined as standardized and machine-readable data resources that contain harmonized annotations and precomputed molecular representations, thereby enabling direct application in downstream analyses such as drug classification, molecular similarity assessment, and drug repositioning.

### Platform architecture and functional modules

The IBDkb platform is organized into six major sections accessible via a unified navigation bar: Homepage, Search, Research Data, Drug Development, Smart Tools, Other, and Help (Fig. 1b). The homepage provides an entry point featuring a global text search bar, links to functional modules, an interactive network visualization, and a data overview panel showing temporal trends and the latest research updates.

The Research Data section contains four modules: Research Articles, Clinical Trials, Therapeutic Strategies, and Pathogenesis. The Drug Development section compiles data on Investigational Drugs, Bioactive Compounds, and Drug Targets. The ‘Other’ section provides supplementary, curated resources, including Experimental Models, Associated Diseases, and Therapeutic Targets. Each module supports structured data display, keyword search, and customizable filtering. The Smart Tools section provides AI-assisted functionalities, including a knowledge acquisition module that retrieves published literature from PubMed Central and performs intelligent content analysis using large language models.

To enhance usability, the search page summarizes the number of matched records within each key module. Several modules support multi-attribute search, including keyword-based queries, conditional filtering, and advanced search. The help page offers a user guide, update logs, and a feedback interface.

## Results

### Overview of curated data in IBDkb

**Figure 2:**
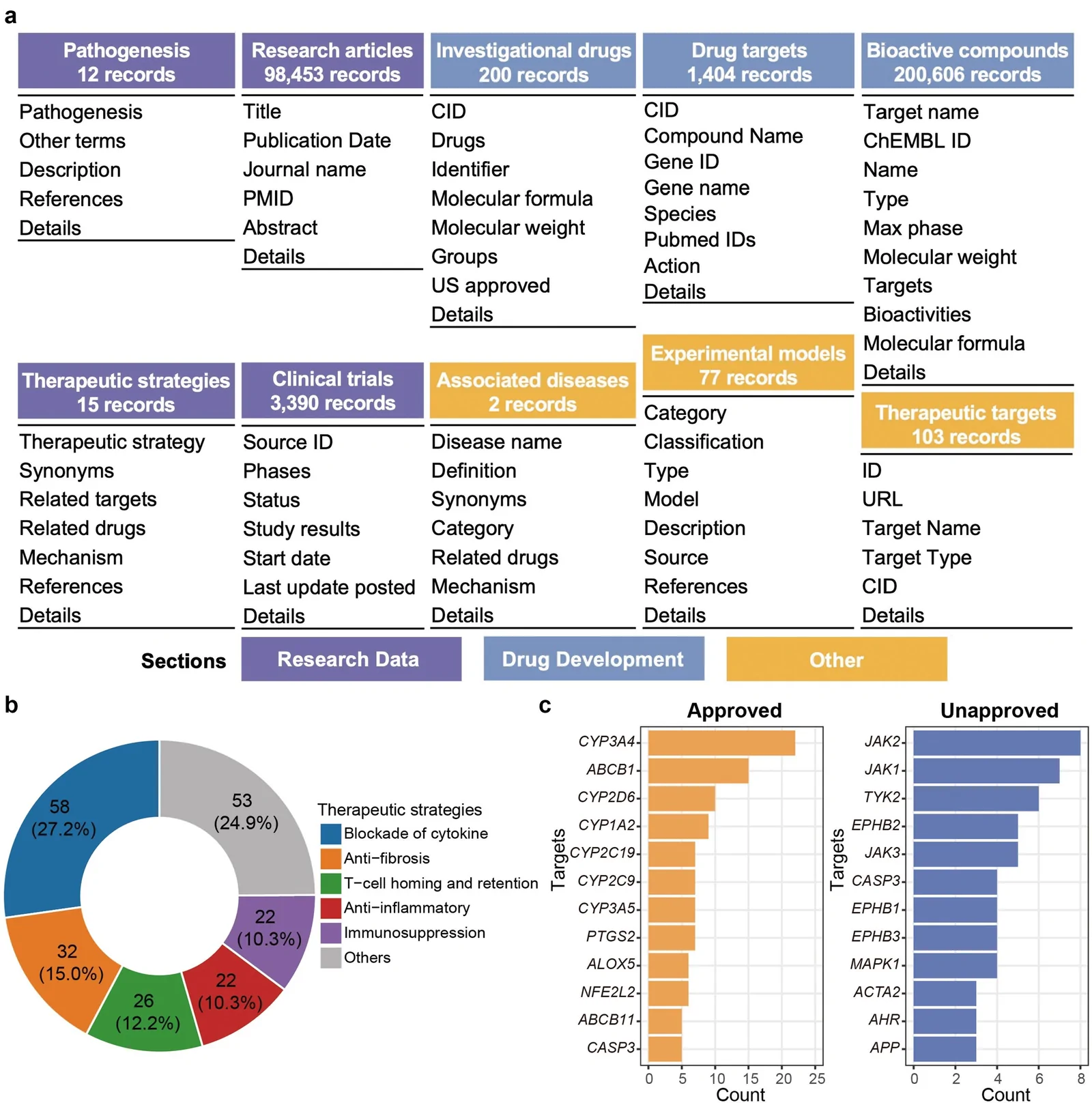
Overview of curated data in IBDkb. (a) Core information displayed in each module of IBDkb. (b) Distribution of therapeutic strategies among investigational IBD drugs. (c) Top 12 drug targets identified in approved and unapproved IBD drugs, respectively. For each group, target frequencies were calculated based on the drug-target associations recorded in the Drug Targets Table, and targets were ranked in descending order according to their occurrence frequency. The 12 most frequently occurring targets in each group are shown.

In total, IBDkb contains 98 453 research articles, 3390 clinical trials, 12 pathogenesis records, 15 therapeutic strategy records, 200 investigational drugs, 1404 drug targets, 200 606 bioactive compounds, 77 experimental models, and 103 IBD-specific therapeutic targets. All data are freely accessible and periodically updated via the IBDkb website (https://www.biosino.org/ibdkb/). Each module provides a concise summary view with key information (Fig. 2a). Detailed information for each record is accessible via a ‘Details’ link.

We summarized the therapeutic strategies of investigational drugs and observed that the majority act through cytokine signalling blockade (27.2%), followed by anti-fibrotic mechanisms (15.0%), modulation of T-cell homing and retention (12.2%), anti-inflammatory effects (10.3%), and immunosuppression (10.3%) (Fig. 2b). Relatively few drugs currently target angiogenesis or stem cells transplantation, indicating that these are underexplored areas. Furthermore, we analysed drug targets of U.S. Food and Drug Administration-approved vs. unapproved IBD drugs. Approved drugs primarily target pathways related to xenobiotic metabolism and transport, inflammatory lipid mediator biosynthesis, and oxidative stress responses, whereas unapproved drugs are mainly enriched for targets involved in JAK-STAT signalling and intestinal epithelial homeostasis (Fig. 2c).

### Platform features and utilities

**Figure 3:**
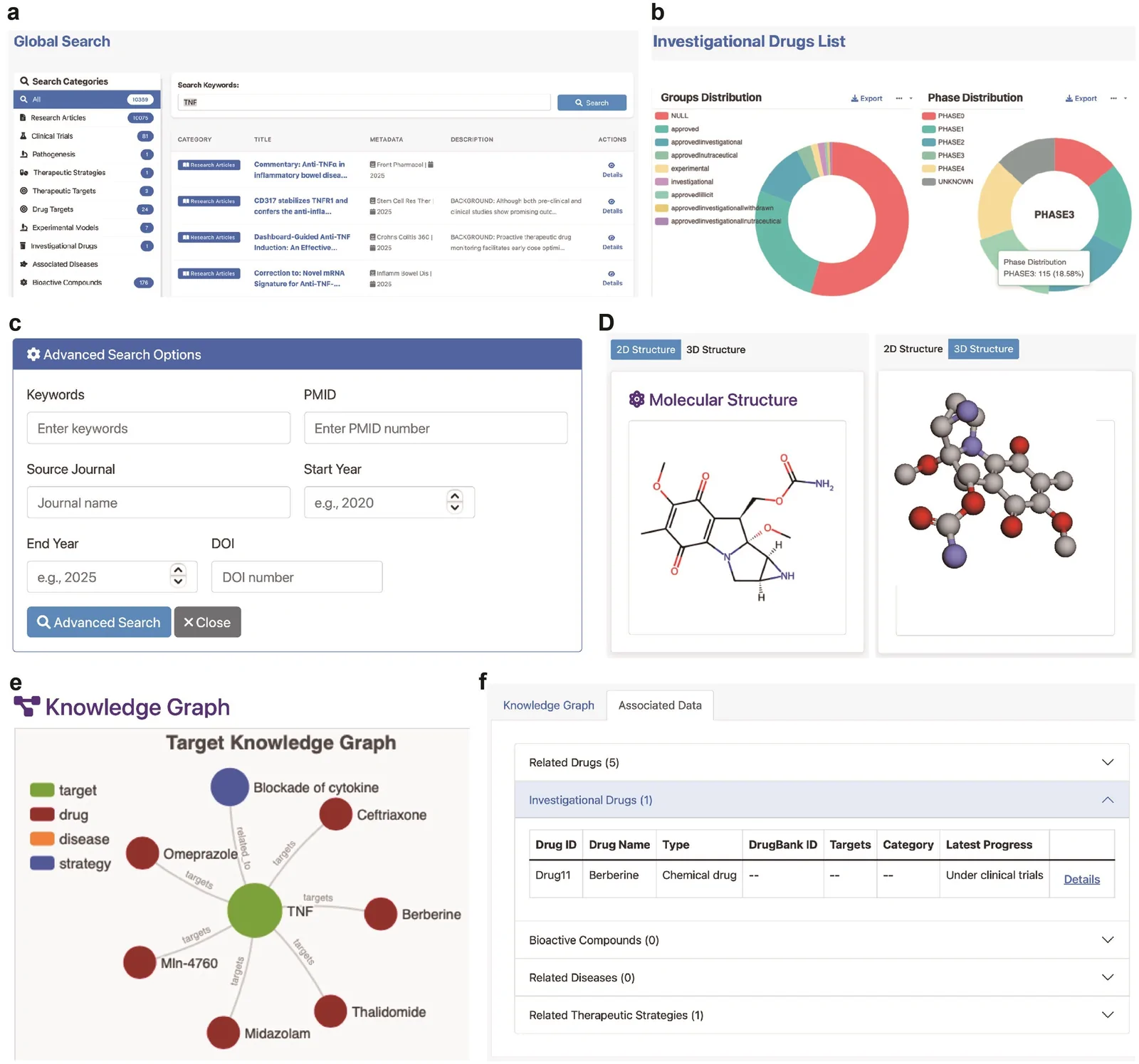
Utility of the IBDkb platform. (a) Categorized display of global search results. (b) Interactive visualization of data distribution within a selected module, exemplified by the Investigational Drugs module. (c) Advanced search functionality within the Research Articles module. (d) 2D and 3D molecular structure visualization, shown using Mitomycin A from the Bioactive Compounds module as an example. (e) Interactive network graph illustrating relationships across associated modules, exemplified by the TNF gene from the Drug Targets module. (f) Corresponding textual information displayed alongside the network, exemplified by the TNF gene from the Drug Targets module.

IBDkb offers robust search and browsing capabilities to facilitate efficient data exploration. A convenient full-text search function allows queries across all textual data, with results organized by module and directly available for download (Fig. 3a). Users can also browse data by module and apply built-in filters to refine search results. Selected modules further incorporate interactive visualizations and advanced search options to support more precise data retrieval (Fig. 3b–c).

To ensure transparency, IBDkb provides direct links to original sources. Pathogenesis and therapeutic strategy records are accompanied by a list of related literature references, so users can obtain more detailed background information from those cited studies. The Drug Development section integrates external resources and visualization tools. Drug-related records include links to relevant clinical trials or literature. The Investigational Drugs and Bioactive Compounds modules provide 2D and 3D molecular structure visualizations (Fig. 3d). Notably, IBDkb highlights cross-module relationships (Fig. S1a). In the detailed view of Investigational Drugs and Drug Targets modules, users can see connections to other modules (e.g. associated clinical trials, literature, or therapeutic strategies) via interactive network graph and text descriptions (Fig. 3e–f).

In the ‘Other’ section, the Experimental Models and Associated Disease modules provide reference links for each entry, enabling users to directly access supporting information. Similarly, the Therapeutic Targets module offers cross-module association details. In addition, IBDkb provides downloadable resources, including curated datasets and AI-ready data comprising MolFormer-based embeddings and molecular fingerprints for drugs and bioactive compounds (Fig. S1b).

### AI-assisted knowledge acquisition module

The Knowledge Acquisition module in the Smart Tools section enhances research capabilities through three main functionalities: literature statistics, literature analysis, and an intelligent conversational interface.

**Figure 4:**
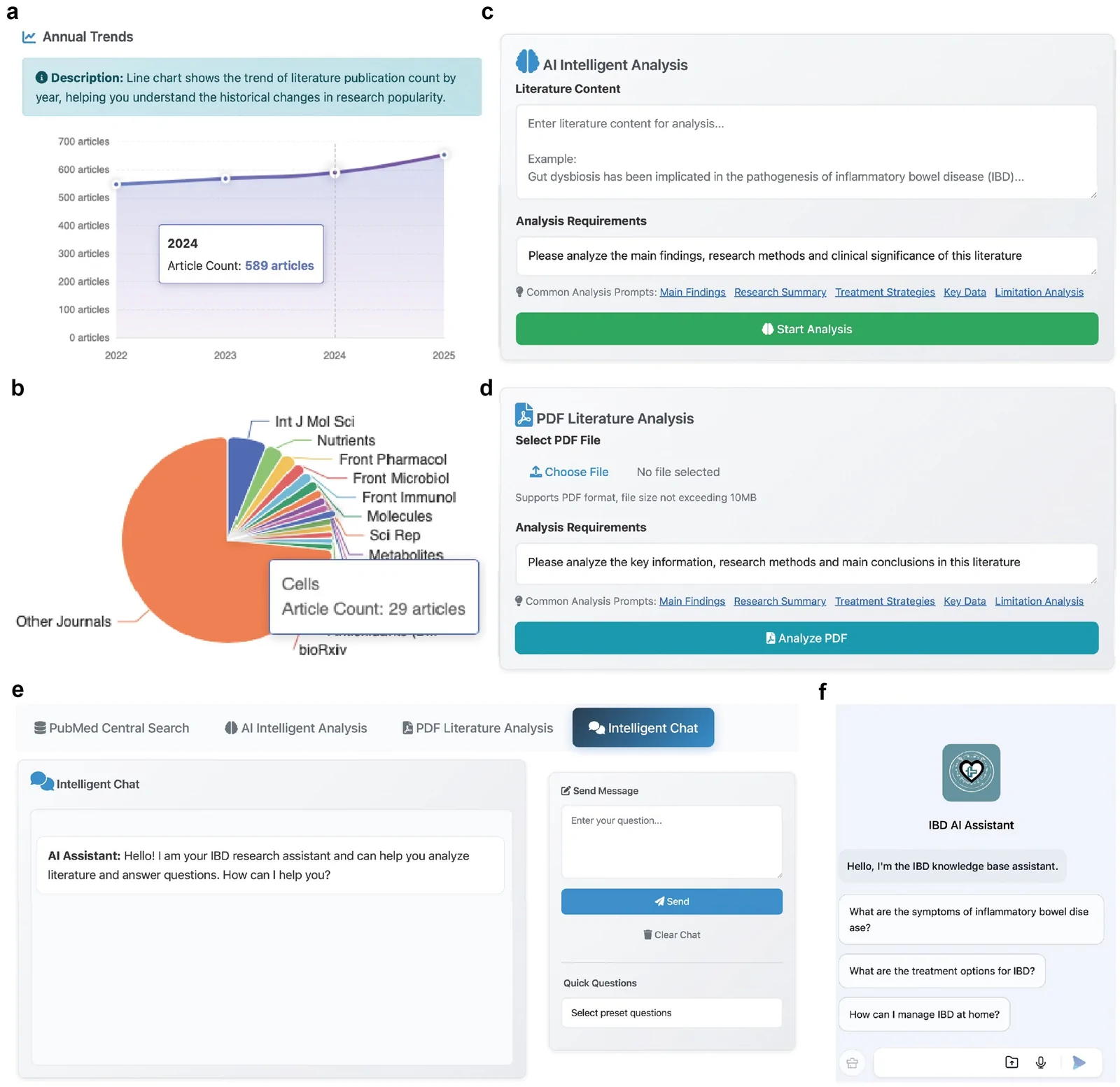
AI-assisted knowledge acquisition in IBDkb. (a) Line chart showing the annual publication trend (2022–2025) for the search term ‘5-Hydroxytryptophan’. (b) Pie chart illustrating the journal distribution of literature in the same search results (2022–2025, ‘5-Hydroxytryptophan’). (c) AI-based intelligent analysis of user-input literature content. (d) AI-based intelligent analysis of uploaded PDF literature files. (e) Intelligent chat function for interactive querying and contextual reasoning. (f) Global AI assistant accessible across the platform.

This module integrates with the PubMed Central for real-time retrieval of the latest literature by keyword, filterable by year range. It provides trend analysis visualizations of publication counts over time and average annual counts (Fig. 4a). A journal distribution visualization shows how results are spread across academic journals (Fig. 4b).

The AI-driven literature analysis component supports both direct text input and PDF upload, enabling intelligent analysis based on user-defined requirements (Fig. 4c–d). To further facilitate knowledge acquisition, the module incorporates an intelligent conversational interface that supports continuous, context-aware dialogue (Fig. 4e). Additionally, a global RAG-based AI assistant is accessible throughout the platform, providing expert-level guidance and enhancing the overall research experience (Fig. 4f). The Knowledge Acquisition module is designed primarily for task-specific literature retrieval, publication trend analysis, and AI-assisted interpretation of user-provided text or PDF files, whereas the global AI assistant serves as a platform-wide conversational interface based on the IBDkb knowledge corpus to support cross-module querying, evidence navigation, and context-aware knowledge exploration.

## Case study: structure-aware drug comparison and repositioning

**Figure 5:**
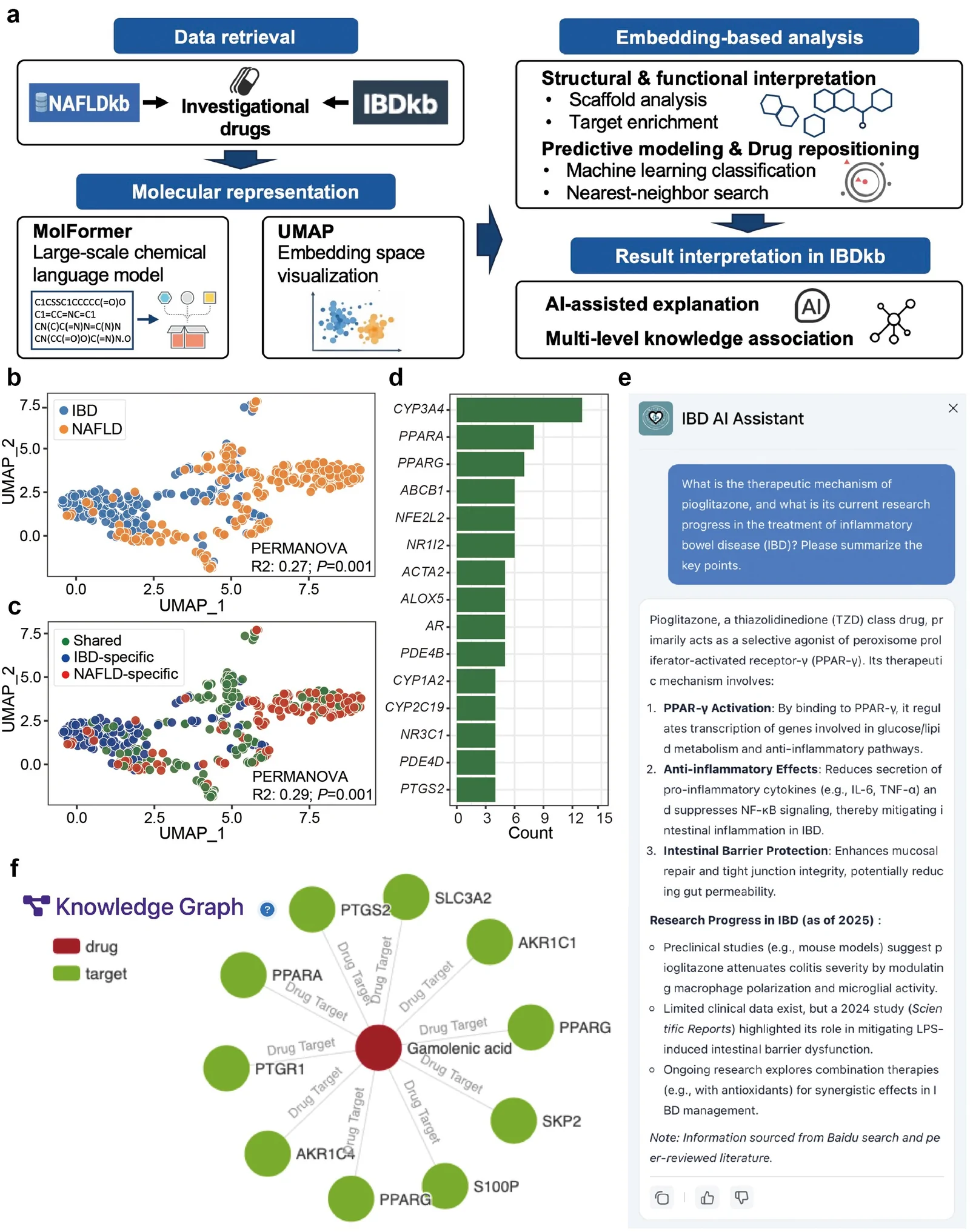
Structure-aware drug comparison and repositioning. (a) The workflow of structure-aware comparative analysis of investigational drugs for IBD and NAFLD based on molecular embeddings. (b) UMAP visualization of MolFormer-derived molecular embeddings for IBD- and NAFLD-associated drugs, with a UMAP-based silhouette score of 0.24. *P* value and *R*-square value were calculated with PERMANOVA by 999 permutations. (c) UMAP projection of the same embedding space annotated by Murcko scaffold categories, including IBD-specific scaffolds, NAFLD-specific scaffolds, and scaffolds shared between the two disease indications, with a UMAP-based silhouette score of 0.024. *P* value and *R*-square value were calculated with PERMANOVA by 999 permutations. (d) Target distribution of drugs associated with the shared scaffold category. Bar plots show the top 15 most frequently occurring targets. Target frequencies were calculated by aggregating the drug-target associations of all drugs containing shared scaffolds, followed by ranking targets in descending order according to their occurrence frequency. Bar plots show the top 15 most frequently occurring targets. (e) Results retrieved using the global AI assistant to query the therapeutic mechanisms of pioglitazone and its current research progress in IBD. (f) Knowledge association network displayed on the detail page of gamolenic acid.

To demonstrate IBDkb’s utility for integrative drug-centric knowledge discovery, we conducted a comparative case study focusing on investigational drugs for IBD and non-alcoholic fatty liver disease (NAFLD) (Fig. 5a). IBD drugs were retrieved from IBDkb, while NAFLD-related drugs were obtained from NAFLDkb [27]. The curated datasets enabled direct input into large-scale language models. Molecular representations were generated using the MolFormer large-scale chemical language model [28], followed by UMAP dimensionality reduction with n_neighbours = 15 and min_dist = 0.1 for visualization (Fig. 5b, Table S2). IBD- and NAFLD-associated drugs showed partially separated spatial distributions in the UMAP space, suggesting that the molecular embeddings captured disease-associated structural differences while retaining overlap between the two drug groups (*R*^2^ = 27%, *P* = 0.001, Fig. 5b).

By transforming heterogeneous drug information into unified embedding representations, we generated AI-ready datasets that support direct integration with downstream machine learning tasks, including classification, similarity analysis, and knowledge discovery. To interpret the structural basis, Murcko scaffold analysis was performed, categorizing scaffolds into IBD-specific, NAFLD-specific, and shared groups (Table S2). Annotation by Murcko scaffold categories revealed scaffold-associated distribution patterns in the UMAP space, although the IBD-specific, NAFLD-specific, and shared scaffold groups showed substantial overlap (Fig. 5c). Permutational multivariate analysis of variance (PERMANOVA) further indicated a significant difference in the overall UMAP distribution among the scaffold categories (*R*^2^ = 29%, *P* = 0.001, Fig. 5c). Target enrichment analysis revealed that shared scaffolds were enriched for targets involved in drug metabolism/transport, inflammation, and immune signalling, such as PPARs, suggesting potential mechanistic overlap (Fig. 5d). Building on these embedding features, five machine-learning classifiers were trained to distinguish IBD drugs from NAFLD drugs. All models achieved robust performance, with five-fold cross-validation mean area under the curve values exceeding 0.93 (Fig. S2a), further confirming structural differences.

Nearest-neighbour analysis in the embedding space explored cross-disease drug repositioning opportunities. Several NAFLD drugs (e.g. LYS006, PF-05221304, and pioglitazone) were identified as closest structural neighbours to multiple IBD drugs, suggesting possible therapeutic transferability (Fig. S2b, Table S3). Conversely, some IBD drugs (e.g. 2’-fucosyllactose, probiotic, and gamolenic acid) showed high structural similarity to NAFLD agents (Fig. S2c, Table S4). These findings indicate that structural proximity can provide a hypothesis-generating signal for cross-disease drug repositioning; however, such similarity should not be interpreted as direct evidence of pharmacological equivalence or clinical efficacy, and the candidate associations require further experimental and clinical validation.

We further leveraged IBDkb’s AI tools to interpret the results. Pioglitazone, structurally proximal to IBD drugs (Fig. S2b), was characterized as a PPARγ agonist with anti-inflammatory and intestinal barrier-protective effects (Fig. 5e) [29]. A global search in IBDkb identified 15 publications on pioglitazone in IBD (Fig. S2d). Although PPARγ has emerged as a novel therapeutic target in IBD [30], existing studies are largely preclinical [31]. In addition, 90 bioactive compound records associated with pioglitazone were retrieved (Fig. S2d, Table S5), providing potential insights for IBD drug development. Gamolenic acid, structurally proximal to NAFLD drugs (Fig. S2c), was linked to 10 drug target records in IBDkb, including PPARs (Fig. 5f), highlighting its potential relevance in NAFLD. This case study demonstrates how IBDkb, integrated with molecular representation models, can facilitate structure-aware drug comparison and support cross-disease repositioning hypotheses.

## Discussion

IBDkb is a comprehensive, therapy-oriented knowledge base and platform for IBD, designed to systematically organize and connect heterogeneous information across drugs, therapeutic strategies, experimental models, clinical trials, and literature. Unlike existing IBD resources that primarily focus on individual omics layers or specific data types [12–19], IBDkb adopts a therapy-oriented and AI-enhanced framework that links drugs, targets, clinical evidence, disease mechanisms, and experimental models within a unified platform. This design enables users to move beyond isolated data retrieval and supports translational research.

Beyond the structure-aware drug repositioning case study, IBDkb can support diverse types of IBD-related scientific research. Researchers can use the platform to retrieve IBD-related literature, track research trends, and summarize evidence for specific drugs, targets, or therapeutic strategies. The integrated clinical trial and investigational drug modules allow users to examine the development status of candidate therapies, compare therapeutic mechanisms, and identify underexplored drug classes or targets. Moreover, cross-module knowledge links facilitate mechanism-driven hypothesis generation and candidate prioritization.

Despite advances in understanding IBD pathophysiology, the gap between mechanistic insights and clinical translation contributes to high preclinical attrition rates in IBD drug development, for which AI-based large-scale data analysis offers promising opportunities to improve efficiency [32]. By harmonizing drug-related information, mechanistic annotations, and clinical trial evidence within a unified framework, IBDkb enables systematic comparisons across therapeutic strategies and disease contexts. This integrative design facilitates applications such as drug repurposing and mechanism-driven trial design, while providing structured and computable background knowledge for AI-assisted drug discovery workflows. IBDkb further offers downloadable AI-ready datasets that enable direct application of machine learning models for drug classification, similarity analysis, and knowledge discovery, lowering the technical barrier for AI-based analyses.

The incorporation of AI-assisted knowledge exploration further enhances IBDkb’s utility. With the rapid advancement of large language models, AI-based tools have demonstrated substantial potential for biomedical knowledge synthesis and reasoning [33]. RAG frameworks, combined with curated domain knowledge, are increasingly applied to tasks such as literature interpretation and hypothesis generation [34]. Compared with traditional query-based databases, the AI assistant embedded within IBDkb enables more flexible and efficient exploration of complex therapeutic evidence, facilitates connections between drug mechanisms and clinical outcomes, and supports hypothesis generation through context-aware interactions.

Despite these strengths, IBDkb also has limitations. The current version primarily relies on published studies and publicly available clinical trial data, necessitating continuous updates. Future extensions will aim to incorporate longitudinal treatment outcomes and multi-omics data associated with therapeutic responses. From an AI perspective, further integration with predictive modelling frameworks and digital twin-based approaches may enable more advanced simulation, outcome prediction, and personalized therapeutic exploration. These developments will further strengthen IBDkb as a dynamic and extensible platform for IBD research and drug development.

## Conclusion

IBDkb is a unique, community-oriented resource that integrates broad, curated IBD data with intuitive visualization and cutting-edge AI-enhanced tools. Freely available without registration, it supports downloadable datasets and welcomes user feedback. By bridging molecular insights with therapeutic development, IBDkb aims to accelerate precision medicine and innovation in IBD research.

## Supplementary Material

baag038_Supplemental_Files

## Data Availability

All the software packages used in this study are open-source and publicly available. All code used and/or analysed in this study are available on GitHub at https://github.com/tjcadd2020/IBDkb.
